# Comparison of the Faecal Microbiota Composition Following a Dairy By-Product Supplemented Diet in Nero Siciliano and Large White × Landrace Pig Breeds

**DOI:** 10.3390/ani13142323

**Published:** 2023-07-16

**Authors:** Viviana Floridia, Letterio Giuffrè, Domenico Giosa, Francesca Arfuso, Francesca Aragona, Francesco Fazio, Cai Chen, Chengy Song, Orazio Romeo, Enrico D’Alessandro

**Affiliations:** 1 Animal Production Unit, Department of Veterinary Sciences, University of Messina, Viale Palatucci 13, 98168 Messina, Italy; viviana.floridia@studenti.unime.it (V.F.); francesca.arfuso@unime.it (F.A.); francesca.aragona@unime.it (F.A.); francesco.fazio@unime.it (F.F.); 2 Department of Chemical, Biological, Pharmaceutical and Environmental Sciences, University of Messina, Viale Ferdinando Stagno D’Alcontres 31, 98166 Messina, Italy; letterio.giuffre@unime.it (L.G.); domenico.giosa@unime.it (D.G.); oromeo@unime.it (O.R.); 3 College of Animal Science & Technology, Yangzhou University, Yangzhou 225009, China; chencai9596@hotmail.com (C.C.); cysong@yzu.edu.cn (C.S.)

**Keywords:** dairy by-product, Nero Siciliano, pigs, metagenomics, faecal microbiota

## Abstract

**Simple Summary:**

The genetic background of the host, together with several other biotic and abiotic factors, including feeding, plays a crucial role in modulating the gut microbiota composition of many animal species. Furthermore, several authors have reported that the microbiota of native pig breeds, such as the Nero Siciliano, reflects distinctive traits that commercial crossbreds have lost. For this reason, the present study shows a comparison of the faecal microbiota composition, following a liquid whey-supplemented diet, in the Nero Siciliano and commercial (Large White × Landrace) pig breeds and the important role of the host genetics in the modulation in faecal microbial composition.

**Abstract:**

The current study compared the faecal microbiota composition of two pig breeds (autochthonous vs. commercial) to understand what happens after the integration of liquid whey in the diet and what the role of the host genetic is. The trial was conducted for 60 days, and the faecal microbiota composition was investigated at three time points, T0, T1 (after 30 days) and T2 (after 60 days) in 30 female pigs (20 commercial crossbred and 10 Nero Siciliano pigs). The animals were divided into four groups (two control and two treatment groups). Generally, in both breeds, *Firmicutes* (51%) and *Bacteroidota* (36%) were the most abundant phylum whereas *Prevotella*, *Treponema* and *Lactobacillus* were the most abundant genera. The two breeds have a different reaction to a liquid whey diet. In fact, as shown by PERMANOVA analysis, the liquid whey significantly (*p* < 0.001) affects the microbiota composition of crossbreeds while not having an effect on the microbiota of the Nero Siciliano. Despite this, in both breeds *Bifidobacterium* and *Ruminococcus* have been positively influenced by liquid whey and they promote intestinal health, improve immunity, increase performance, and feed efficiency. In conclusion, the integration of liquid whey had a different effect on the Nero Siciliano and crossbred pig breeds, emphasizing the importance of the host genetic profile in determining the faecal bacterial composition.

## 1. Introduction

Today, the scientific community fully agrees on the importance of gut microbiota in the health status and welfare of swine [1,2]. Therefore, a deep knowledge of the taxonomic composition, abundance, and diversity of microbial communities living in the intestine can provide important information that would allow us to promote animal health [3,4]. Generally, in the gut microbiota of pigs, there are several bacteria and other microorganisms whose relative abundance changes during the host growth [5,6], mainly from the post-natal to weaning periods [7,8,9,10]. During the entire life of swine, the composition is affected by environmental [11] and feeding factors [12,13].

Both the diet formulation, including the integrations of feed and the typology of feed administration, such as liquid feeding [14], affect the taxonomy of gut microbiota [12,13,15]. Moreover, several authors have suggested that gut microbiota composition influences feed efficiency and represents a crucial parameter of swine production [3,16,17,18].

It has been demonstrated that the composition of gut microbiota is deeply conditioned by host genetics [11,12,13,19] and important differences between microbiomes of commercial and autochthonous pig breeds, such as South African Windsnyer-type indigenous pigs [12], Iberian pigs [13], Chinese Bama minipigs [19], Tibetan pigs [19], and Jinhua pigs [20,21], have been reported. These differences were believed to be related to the typical rustical traits characterizing the autochthonous breeds and this assumption was also supported by Giuffrè et al. [22] and Tardiolo et al. [23], studying the taxonomic composition of faecal microbiome of the Nero Siciliano (NS) pig, an important autochthonous breed reared in Sicily, Italy [24]. The NS breed forms part of Sicily’s cultural and historic heritage and has many peculiar characteristics, such as great potential to adapt to difficult conditions, both climatic and environmental, the ability to resist disease and a marked adipogenic capacity [25,26,27]. The adaptive traits and the peculiar characteristics are almost lost in the commercial pigs as a consequence of the severe genetic improvement programs to which they have been subjected [28].

In recent years, there has a new challenge involving the valorization of Agri-Food Waste (AFW) originating throughout the whole food chain, from production to industrial processing (FAO https://www.fao.org/home/en) (accessed on 10 April 2023). AFW can be used both as bioactive compounds, such as phenolic compounds [29], and as food ingredients for the feed industry, as they pose no risk to animal health [30,31]. An important AFW is liquid whey, a dairy by-product widely used in animal nutrition to reduce the environmental impact as it has a positive effect on farm income [32]. Liquid whey is considered a nutritionally beneficial food additive because it is rich in proteins, vitamins, and minerals [33] and like all liquid foods, has a beneficial effect on protein and fat digestibility and mineral absorption, allowing better growth of swine [34,35]. Moreover, Demecková et al. [14] have shown that the administration of fermented liquid feed to farrowing sows influences positively their gut microbial compositions with a significant improvement in the colostrum quality and consequently in the health of piglets. Interestingly, liquid whey affects the faecal microbiota composition via its high lactose content (5%) which is fermented by intestinal lactic acid bacteria (LAB) into lactic acid [15]. This carboxylic acid reduces the intestinal pH and consequently improves intestinal health; in fact, a low intestinal pH prevents the growth of pathogenic bacteria, such as *Salmonella* and *Escherichia coli* [36].

The aims of this study were the comparison of the faecal microbiota composition, following a liquid whey-supplemented diet, in Nero Siciliano and commercial (Large White × Landrace) pig breeds and to establish whether the genetics of the host influence the faecal microbial composition.

## 2. Materials and Methods

### 2.1. Animals, Diets, and Experimental Design

The study was approved by the Animal Experiment Ethics Committee of Messina University (Authorization number 055_2021) according to the European guidelines for the care and use of animals in research (Directive 2010/63/EU 2010). All procedures were carried out according to relevant guidelines and regulations.

The trial was conducted for a total of 60 days, from day 58 until day 118 of life, with an adaptation period to the diet of 15 days.

A total of 30 female pigs, 20 commercial crossbred (CB), Landrace × Large White, and 10 Nero Siciliano (NS) pigs, homogeneous for body weight (average initial body weight of 19.4 ± 1.92 kg), age (58 ± 2 days), and breeding system, was divided into four groups: two control and two treatment groups, as reported in Table 1.

The two control groups (crossbred, CB_CTRL; Nero Siciliano, NS_CTRL) were fed with a pellet complete feed (same batch of feed for the entire trial) rationed based on 3% of the live weight, and the two treatment groups (crossbred, CB_TRT; Nero Siciliano, NS_TRT) received the same diet integrated with fresh liquid whey (5% carbohydrate, 0.8% proteins, 0.6% fat, 93% water, and 0.6% ash) at the level of 1.5 L/day/pig for 8 weeks. The two diets were isoenergetic and isoproteic and the ingredients and composition of the complete feed are reported by D’Alessandro et al. [26].

All pigs were kept in individual pens with nipple waterers and stainless-steel feeders. The feed was administered individually twice a day, in the morning and in the afternoon, and liquid whey was fed separately from the formula feed, in the late afternoon, using a wet feeder.

The animals had no gastrointestinal diseases or any antibiotic exposure prior to the study. None of the animals had access to the outside and they were exposed to a natural photoperiod and natural environmental temperature, as reported by D’Alessandro et al. [26]. Thermal and hygrometric records were logged inside and outside the pen for the whole study by means of a data logger (Gemini, London, UK).

### 2.2. Faecal Sample Collection and 16S Sequencing

Faecal samples were collected directly from the rectal ampulla of each pig (Table 1) at different time points, starting from the initial condition (T0) followed by two time points when they were collected after 30 (T1) and 60 days (T2), respectively.

A total of 90 stool samples was immediately stored in aliquots of ~400 mg using the OMNIgene^®^•GUT tubes according to the manufacturer’s instructions. Illumina MiSeq^®^ paired-end (2 × 300 bp) sequencing (Illumina, San Diego, CA, USA), targeting the V3-V4 region of the 16S rRNA gene, was performed by an external service provider (Eurofins genomics, Ebersberg, Germany).

The obtained raw data were deposited in the NCBI Sequence Read Archive (SRA) under the accession code PRJNA911158.

### 2.3. Sequence Processing and Analysis

Illumina sequencing reads were processed by the QIIME2 v. 2022.2 pipeline [37]. The amplicon sequence variant (ASVs) feature table was constructed and denoised using the DADA2 pipeline [38] with default settings. According to Henderson et al. [39], a cut-off of 75%, 87%, and 95% identity was used to taxonomically classify the microbial reads at the phylum, family, and genus level, respectively. A SILVA reference database version 138 [40] was used for taxonomic classification. Phylum, families, or genera that had a relative abundance below 0.1% were grouped as “others”.

### 2.4. Alpha Diversity, Beta Diversity, Differential Analysis, and Prediction of Microbial Functions

The determination of diversity within the faecal bacterial community was performed using RStudio software. The phyloseq R package [41] was used to compute alpha and beta diversity values. At the genus level, the alpha diversity was evaluated using three measures (observed richness, Chao1index, and Shannon index) and the box plot, based on the Shannon index, was generated using a ggplot2 [42] package.

Beta diversity was computed using the Bray–Curtis distance method and plotted with a principal component analysis (PCoA) using a ggplot2 R package [42].

Differential analysis was performed using the DESeq2 package in R [43] and the differences between breeds and diets were analyzed in separate models (Table 2) as reported by López-García et al. [13]. The visualization of detected differences was made using the EnhancedVolcano package in R [44].

### 2.5. Statistical Analysis

To evaluate the distribution of alpha diversity, based on the Shannon index, a one-way nonparametric Wilcoxon test was used for diet and breed variables, while the nonparametric Kruskal–Wallis test was employed for the time variable.

The PCoA, which evaluates the differences between samples based on the Bray–Curtis distance, was assessed through permutational multivariate analysis of variance (PERMANOVA) with 999 permutations using the vegan package [45].

The differences produced during the differential analysis were determined by a Wald test *p*-value and were considered statistically significant using a false discovery rate (FDR) cut-off of 0.05 and a fold-change (FC) higher than 1.5 or lower than −1.5 (i.e., |log2FC|> ±0.59). Significance was determined at *p* ≤ 0.05.

## 3. Results

### 3.1. Taxonomy Classification

A total of 6.543.707 high-quality (phred-score > 20) sequences, detected in both conditions of diets (CTRL and TRT) of the CB and NS, was used for taxonomic recognition. The sample metadata, the denoising statistics, the taxonomy classification, and the relative abundance of each taxonomic level are reported in Supplementary Materials (Table S1). The overall microbiota composition is reported in Table 3.

The percentage of relative abundances at the phylum level for the CTRL and TRT of both breeds is reported in Supplementary Materials (Table S1). All detected phyla were found in the TRT group of the CB breed, and in the CTRL group of the NS breed. By contrast, in the CTRL group of the CB no *Deferribacterota* was found while *Verrucomicrobiota* was not present in the CTRL group of the NS.

**Figure 1:**
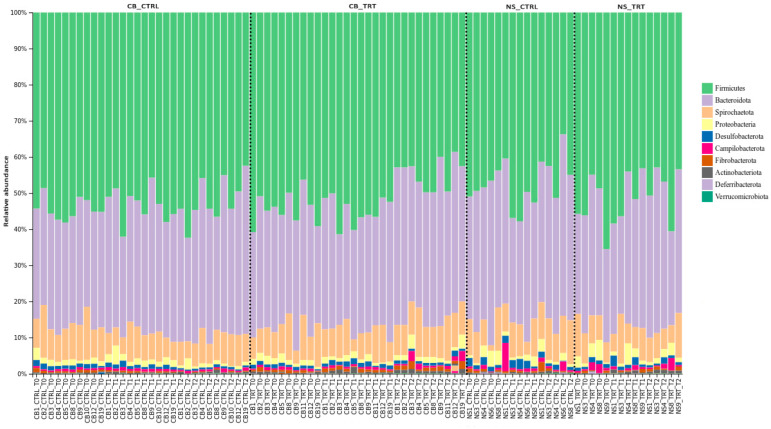
Taxonomy bar plot illustrating the relative abundance at phylum level.

The taxonomy bar plot (Figure 1) shows graphically the abundance of each bacterial phylum across all examined samples. *Firmicutes* and *Bacteroidota* were the most abundant phyla in both CTRL and TRT groups, but it is clear that in most samples of both breeds and diet conditions, *Firmicutes* prevail over *Bacteroidota*. However, in one sample for each group (CB19_CTRL_T2, NS6_CTRL_T2 and NS9_TRT_T1) the profile was different as the *Bacteroidota* were the most abundant phylum detected (Figure 1).

We found that the *Firmicutes: Bacteroidota* ratio, in a few samples belonging to the CB, was different in the liquid whey diet at T2. In fact, in this case *Bacterodoita* were the most abundant phylum compared to *Firmicutes*.

At the family level, we identified 51 microbial families in the CTRL groups of the CB, 55 families in the TRT of the CB, 48 families in the CTRL of the NS and 45 families in the TRT of the NS (Table 3). In particular, *Prevotellaceae*, *Spirochaetaceae*, *Lactobacillaceae*, *Lachnospiraceae*, *Oscillospiraceae*, *Ruminococcaceae*, and *Rikinellaceae* were the most abundant families in both groups.

At the genus level, we identified 106 microbial genera in the CTRL groups of the CB, 110 genera in the TRT of the CB, 109 genera in the CTRL of the NS and 104 genera in the TRT of the NS (Table 3). The three most abundant genera are reported in Table 4.

The genera *Alistipes*, *Actinobacillus*, *Paludicola*, *Angelakisella*, *Ruminiclostridium*, *Acinetobacter*, *Prevotellaceae_YAB2003_group*, and *Herbinix* were not detected in both groups (CTRL and TRT) of the CB and are also absent from one of the two groups belonging to the NS breed (Table S1). While *Bacteroidales_BS11_gut_group*, *Bacteroidales_UCG-001*, *CAG-873*, *Herbinix*, *Olsenella*, *Mailhella*, *Lachnospiraceae_FCS020_group*, *Prevotellaceae_YAB2003_group*, *Fusobacterium*, *Chloroplast*, *Aminicenantales*, *Cyanobium_PCC-6307*, *Escherichia-Shigella*, *Anaerosporobacter*, *JS1*, and *Mitsuokella* were not detected in either group of the NS and were also absent from at least one of the two groups belonging to the CB (Table S1).

### 3.2. Alpha Diversity, Beta Diversity, Differential Analysis, and Prediction of Microbial Functions

The alpha diversity, based on the observed richness, Shannon and Chao1 index, revealed the richness within samples at the genus level (Table S2). Figure S1, in the Supplementary Materials, shows the quantitative difference between the two diet conditions (CTRL vs. TRT) and within three time points (T0, T1, T2) in the CB breed while Figure S2, in the Supplementary materials, shows the same variables but in the NS breed. The differences were evaluated through a Wilcoxon test for the diet while for the time variable, a Kruskal–Wallis test was used. These tests revealed no statistically significant quantitative differences except for the variable time of the CB breed suggesting that over time the microbial richness mayalter.

**Figure 2:**
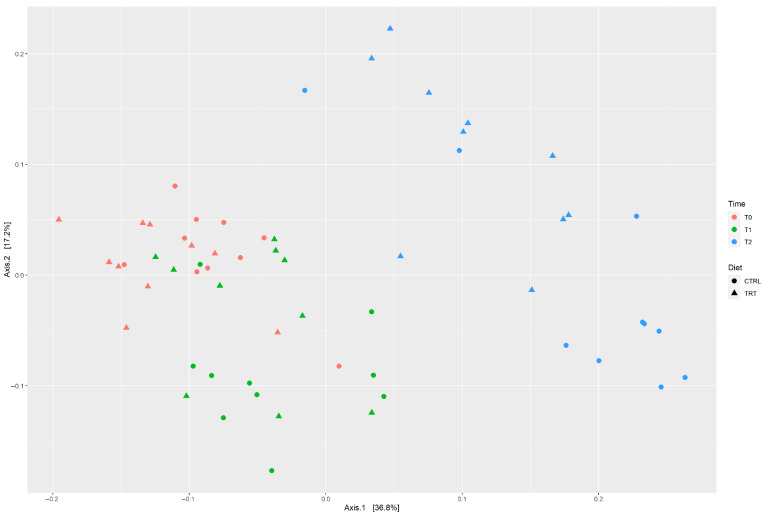
PCoA plot depicting the distribution of two diet groups (CTRL vs. TRT), belonging to CB breed, at three time points using the Bray–Curtis distance.

**Figure 3:**
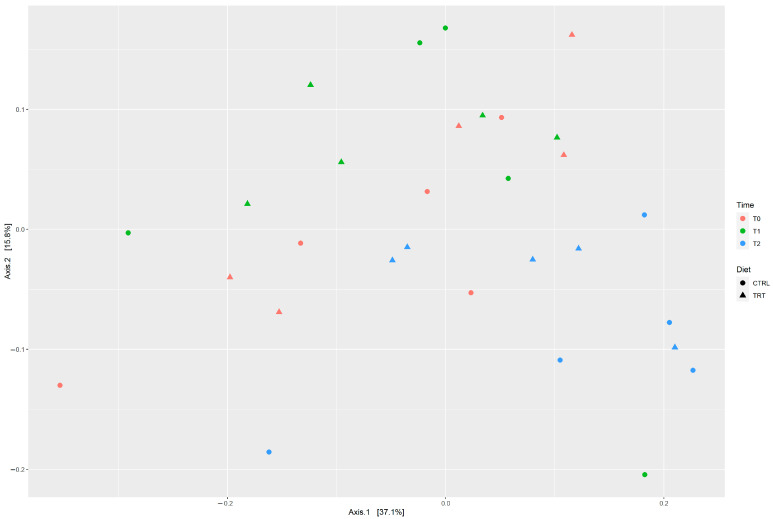
PcoA plot depicting the distribution of diet groups (CTRL vs. TRT), belonging to Nero Siciliano breed, at three time points using the Bray–Curtis distance.

The beta diversity, based on the Bray–Curtis distance method, was used to measure the dissimilarity between samples. In the PCoA plot, the two breeds displayed different behaviors towards diets; the distribution of the CB and NS is depicted in Figure 2 and Figure 3, respectively. For each breed, it is possible to visualize the behavior of samples during the three times (T0, T1, T2) and with different diets. The results of PERMANOVA analysis are reported in Table 5. Interestingly, the microbial communities of the CB were affected by both time and diet (*p* < 0.003 and *p* < 0.001, respectively) whereas in the NS, the PERMANOVA analysis showed that the diet did not affect the microbiota composition although changes may occur over time (*p* < 0.004; Table 5).

The differential analysis was made with DESeq2 and differences between breeds and diets were analyzed in separate models (Table 2).

**Figure 4:**
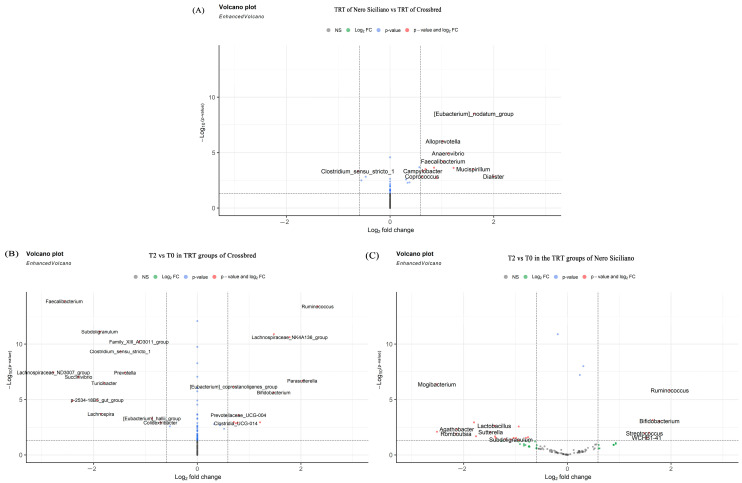
Volcano plots showing significant differences found at genus level between TRT groups of two breeds (NS vs. CB) (**A**), in the liquid whey diet at T2 vs. T0 in CB (**B**) and NS (**C**). Red dots represent genera with an adjusted *p*-value below the threshold 0.05 and ±1.5 for FDR and FC, respectively.

The volcano plot (Figure 4) showed the significant differences, at the genus level, between the TRT groups (NS vs. CB; A) and in the liquid whey diet at T2 vs. T0 both in the CB (B) and NS (C).

Analyzing the differences between the treatment groups of both breeds (Figure 4A), we found that the *[Eubacterium]_nodatum_group*, *Alloprevotella*, *Anaerovibrio*, *Faecalibacterium*, *Campylobacter*, *Coprococcus*, *Mucispirillum*, and *Dialister* were the most predominant in the NS breed while *Clostridium_sensu_stricto_1* was the most abundant in the CB.

In addition, we observed that, under the liquid whey supplementation (TRT group), the bacterial communities can change over time and induce the growth of beneficial bacteria such as *Ruminococcus* and *Bifidobacterium*. These two genera were most prevalent at T2 in both pig breeds (Figure 4B,C).

In addition, analyzing the differences between T2 vs. T0 in the treatment group of the CB (Figure 4B), we found that the *Lachonospiraceae_NK4A136_group*, *[Eubacterium]_coprostanoligenes_group*, *Parasutterella*, *Prevotellaceae_UCG-004*, and *Clostridia_UCG-014* were prevalent at T2 while *Faecalibacterium*, *Subdoligranulum*, *Family_XIII_AD3011_group*, *Lachnospiraceae_ND3007_group*, *Succinivibrio*, *Turibacter*, *p-2534-18B5_gut_group*, *Lachnospira*, *[Eubacterium]_halli_group*, and *Colidextribacter* were less abundant at the same time point and consequently were higher at the begging of the administration.

As regards the differences between T2 and T0 in the treatment group of the NS (Figure 4C), *Streptococcus* and *WCHB1-41* were more prevalent at T2, while *Mogibacterium*, *Agathobacter*, *Romboutsia*, *Sutterella*, and *Subdoligranulum* were less prevalent at T2.

## 4. Discussion

Today, it is clear that the genetic background of the host plays a crucial role in the modulation in abundance of gut microorganisms [11,19].

Several authors have reported that the microbial profiles differ between autochthonous and commercial breeds [12,13,21]. The main comparisons have been conducted between autochthonous breeds, such as South African Windsnyer-type indigenous pigs [12], Jinhua pigs [20,21], Iberian pigs [13], Nero Siciliano pigs [22], and commercial crossbred [12] and/or purebred lines such as the Large White [11] and Duroc [13].

The predominant phylum in both CB groups was *Firmicutes* (53%) followed by *Bacteroidota* (35%), and our results are in accordance with other research [12,15,46]. Although *Firmicutes* were generally more abundant than *Bacteroidota*, a few samples, belonging to the TRT group at T2 of the crossbred, showed a higher prevalence of *Bacteroidota*. This prevalence could be linked to a liquid whey diet [46] because this phylum is generally associated with the piglets’ suckling period [47].

Considering all phyla identified in the NS, *Firmicutes* (47% for CTRL and 51% for TRT) prevailed over *Bacteroidota* (38% for CTRL and 35% for TRT) and this agrees with previous metagenomic analysis in the NS [22] and in other autochthonous breeds, such as African Windsnyer-type indigenous pigs [12], Jinhua pigs [20,21], and Iberian pigs [13]. Several studies reported that the predominance of *Firmicutes* over *Bacteroidota* has been associated with a predisposition to accumulating excess body fat. In fact, the Nero Siciliano as well as other autochthonous pig breeds, including South African Windsnyer-type indigenous pigs [12] and Jinhua pigs [20,21], has an excellent body fat deposition capacity [24,48,49,50,51]. This capacity is typical of autochthonous breeds [12,52,53]. Yang and co-workers [54] have reported that the gut microbiota is a major contributor to adiposity in pigs, and as reported by Luo et al. [52] the microbiota of the Landrace (lean type) has higher diversity and density than Erhualian pigs (obese type). Finally, an association between a predisposition to the accumulation of body fat and a prevalence of *Firmicutes* compared to *Bacteroidota* was suggested in a microbiome survey focused on the marine carnivorous species *Neophoca cinerea* (fat Australian sea lion) [55]. This hypothesis is further supported by molecular pathways linked to fatty acid metabolism detected in the microbiome of the NS [22] and described in high-fatness pigs [56] and obese children [57].

An interesting result obtained in our studies regards the beta diversity analysis in which the two breeds have different reactions to the diets over a specific time period.

Particularly, in the NS, the dietary groups (CTRL and TRT) do not separate. We could hypothesize that the fact that food administration does not statistically (*p*-value = 0.199; Table 5) affect the microbial composition of the Nero Siciliano could be related to its adaptive traits. In fact, this breed has always adapted to all situations [25,26,27], for example to difficult environmental and climatic conditions. Several authors have reported that the host genetics seem to play a key role in the microbial resilience of the host’s microbiota [58,59,60,61], and this could be considered also to be the case for the Nero Siciliano breed.

On the contrary, in the crossbred, the liquid whey diet significantly affects the faecal microbiota composition (*p*-value = 0.003; Table 5).

Generally, thanks to PERMANOVA results (Table 5), during the time from T0 to T2, the microbiota composition changes in both breeds. The main changes happen in the treatment groups; in fact, we have investigated, with differential analysis, which genus differs from the beginning to the end of the trial (T2 vs. T0; Figure 4). As reported by Kobayashi et al. [15], liquid whey stimulates the growth of beneficial bacteria such as *Ruminococcus* and *Bifidobacterium*, and at the end of the trial in both breeds these two genera were most abundant compared to the beginning (Figure 4B,C). In this context, it is interesting to note that despite the fact that the diet had no significant effect on the NS, at T2 the two genera were present.

The *Ruminococcus* belongs to the *Ruminococcaceae* family and to the *Firmicutes* phylum; its presence is related to a high-energy-density diet such as liquid whey [62]. Some beneficial species belonging to this genus ferment fiber to produce short chain fatty acids (SCFAs) such as acetate, butyrate, and propionate [63,64]. The butyric acid produced is positively correlated with anti-inflammatory effects [65,66] and promotes intestinal health [67,68].

The *Bifidobacterium* belongs to the *Bifidobacteriaceae* family and to the *Actinobacteriota* phylum, has a probiotic effect and can also reduce the levels of pro-inflammatory molecules [69]. In fact, its presence has helped to maintain the integrity of the gut barrier to limit the occurrence of pathogen bacteria [36,70,71].

Generally, as reported by Kobayashi et al. [15], the beneficial effect of liquid whey is related to its high lactose content which is converted into (S)-lactate (lactic acid). The lactic acid reduces the intestinal pH preventing the overgrowth of pathogenic bacteria, such as *Salmonella* and *Escherichia coli* [36]. In addition, Kobayashi et al. [15] have also reported that liquid whey is rich in lactobacilli but in our results (Table 4), although the *Lactobacillus* genus showed a relative abundance which tends to increase in the TRT group of both breeds, this was not statistically significant. It can be explained because, as demonstrated by Ohashi et al. [72], the lactobacilli present in liquid whey do not multiply in the guts of pigs but exert their beneficial effects by interacting with indigenous lactobacilli. In our study, another explanation may be due to the fact that the beneficial effect of liquid whey is major in the early stage of growth [15] and this has also been reported in humans because the probiotic integration may have a greater impact in younger infants than in older infants [73]. Moreover, among the most abundant genera (Table 4) we found *Prevotella* (*Prevotellaceae* family) and *Treponema* (*Spirochaetaceae* family). Our results agree with those reported by Ramayo-Caldas et al. [74] who showed that in pigs there are two large microbial enterotypes: PEB, in which belongs *Prevotella,* and PEA, in which belongs *Treponema*. Both genera are able to digest fiber [5,67,74] and produce SCFAs which are associated with beneficial effects in the gastrointestinal tract.

## 5. Conclusions

The current study compared the changes in the faecal microbiota composition of two different breeds after a liquid whey supplement diet.

In fact, on one hand, in the analysis of the beta diversity of the Nero Siciliano, the liquid whey diet does do not have a statistically significant effect; on the other hand, it has an important effect on the modulation in faecal microbiota of commercial pigs. Despite this, in both breeds *Bifidobacterium* and *Ruminococcus* have been the principal genera positively influenced by the liquid whey diet and they promote intestinal health, improve immunity, increase performance, and feed efficiency.

In conclusion, the integration of liquid whey had a different effect on Nero Siciliano and crossbred pig breeds, emphasizing the importance of the host genetic profile in determining the faecal bacterial composition.

## Figures and Tables

**Table 1:** Number of animals for each group.

	CTRL	TRT
Crossbred (CB)	10	10
Nero Siciliano (NS)	5	5

**Table 2:** DESeq2 differential abundance models for each contrast.

Contrast	Model Design
Breed contrast	Diet + Breeds
Interaction effects in crossbred	Diet + Time + Diet: Time
Interaction effects in Nero Siciliano	Diet + Time + Diet: Time

**Table 3:** Overall microbiota composition showing the number of phyla, families, and genera found in this study.

	Phylum (75% ^1^)	Family (87% ^1^)	Genera (95% ^1^)
CB_CTRL ^2^	9	51	106
CB_TRT ^2^	10	55	110
NS_CTRL ^3^	9	48	109
NS_TRT ^3^	10	45	104

^1^ percentage of identity used to classify the ASVs at different taxonomic levels; ^2^ CB: crossbred; ^3^ NS: Nero Siciliano.

**Table 4:** The 3 most abundant genera detected in control (CTRL) and treatment (TRT) groups in both breeds.

Phylum	Family	Genus	Mean of Relative Abundance			
			CB_CTRL	CB_TRT	NS_CTRL	NS_TRT
*Bacteroidota*	*Prevotellaceae*	*Prevotella*	6.56%	7.23%	9.86%	9.11%
*Spirochaetota*	*Spirochaetaceae*	*Treponema*	5.59%	6.43%	7.33%	5.86%
*Firmicutes*	*Lactobacillaceae*	*Lactobacillus*	4.85%	5.76%	5.25%	6.04%

**Table 5:** PERMANOVA statistics obtained in this study.

	Variable	F Statistics	R^2^	*p*-Value
Crossbreds	Diet	5.11	0.05	0.003 *
	Time	15.99	0.34	0.001 *
Nero Siciliano	Diet	1.38	0.04	0.199
	Time	2.82	0.17	0.004 *

* *p* < 0.05.

## Data Availability

Data supporting the results of this study have been deposited in the Sequence Read Archive database under the following study accession number SUB12355841 associated with the BioProject ID PRJNA911158.

## Supplementary Materials

The following supporting information can be downloaded at: https://www.mdpi.com/article/10.3390/ani13142323/s1, Figure S1: Box plots with different genus richness (Shannon index) in all CB samples in different feed conditions and time points; Figure S2: Box plots with different genus richness (Shannon index) in all NS samples in different feed conditions and time points; Table S1: 16S rRNA raw data; Table S2: Alpha diversity (observed richness, Shannon and Chao1 index) in richness within samples at genus level.
