# Supplementary material for: Comparison of the Faecal Microbiota Composition Following a Dairy By-Product Supplemented Diet in Nero Siciliano and Large White × Landrace Pig Breeds
Source: Animals (Basel). 2023 Jul 16;13(14):2323. doi: 10.3390/ani13142323 (PMC10376647; doi:10.3390/ani13142323)

Figure S1. Box plots with different genus richness (Shannon index) in all CB samples in different feed conditions and time points.

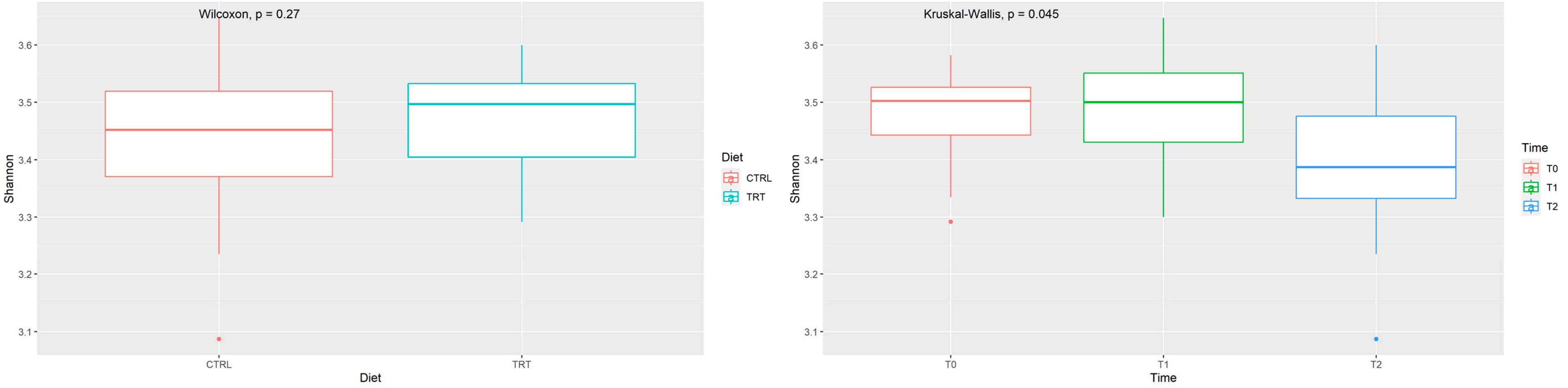

Figure S2. Box plots with different genus richness (Shannon index) in all NS samples in different feed conditions and time points.

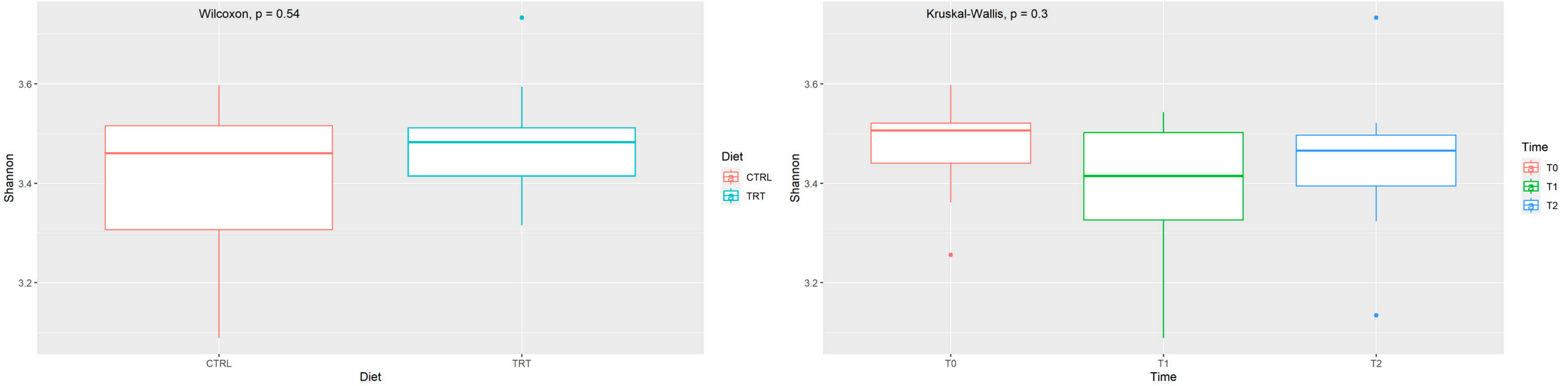

Supplement: Supplementary file 1 [file animals-13-02323-s001.zip › Figures 1 and 2.pdf]
